# Redox Signaling in Plant Heat Stress Response

**DOI:** 10.3390/antiox12030605

**Published:** 2023-03-01

**Authors:** Stefania Fortunato, Cecilia Lasorella, Nunzio Dipierro, Federico Vita, Maria Concetta de Pinto

**Affiliations:** Department of Biosciences, Biotechnology and Environment, University of Bari Aldo Moro, 70121 Bari, Italy

**Keywords:** reactive oxygen species, heat stress, antioxidant, redox signaling, thermotolerance

## Abstract

The increase in environmental temperature due to global warming is a critical threat to plant growth and productivity. Heat stress can cause impairment in several biochemical and physiological processes. Plants sense and respond to this adverse environmental condition by activating a plethora of defense systems. Among them, the heat stress response (HSR) involves an intricate network of heat shock factors (HSFs) and heat shock proteins (HSPs). However, a growing amount of evidence suggests that reactive oxygen species (ROS), besides potentially being responsible for cellular oxidative damage, can act as signal molecules in HSR, leading to adaptative responses. The role of ROS as toxic or signal molecules depends on the fine balance between their production and scavenging. Enzymatic and non-enzymatic antioxidants represent the first line of defense against oxidative damage and their activity is critical to maintaining an optimal redox environment. However, the HS-dependent ROS burst temporarily oxidizes the cellular environment, triggering redox-dependent signaling cascades. This review provides an overview of the redox-activated mechanisms that participate in the HSR.

## 1. Introduction

In recent decades, along with natural causes, anthropic activities have brought about an alarming trend of global warming, which is driving drastic changes in our climate, resulting in more frequent and intense heat waves and causing substantial losses to agricultural production [1,2,3]. Plants can be exposed to different forms of heat stress (HS), such as heat shock, which is an increase in temperature of 10–15 °C above ambient for a short period (from several minutes to a few hours) or prolonged warming that consists in exposing plants to a modest increase in temperature (2–5 °C above ambient) for several days or weeks [4,5]. HS affects plant growth, development, and reproduction at morphological, physiological, and molecular levels [6], even though plants can use different strategies to combat these two types of HS [7].

One worrying consequence of temperature rise is how it severely limits crop productivity. HS occurring during gametogenesis causes morphological abnormalities in pollen and ovary ultrastructure and viability, thus affecting fertilization efficiency; moreover, HS impacts the duration of grain filling, the availability of assimilates, and the enzymes of starch metabolism while also altering the abscisic acid/ethylene ratio [8,9,10]. Like other alterations in environmental conditions, such as cold, drought, flooding or nutrient deficiency, heat directly affects plant growth and modifies plant relations with pathogens, thus strongly impacting crop yield [11]. As occurs in many other biotic and abiotic stresses [12,13,14], under HS, several biochemical and physiological processes are altered, including water potential, transpiration, nutrient uptake and transport, cell division and differentiation, respiration, and photosynthesis [15,16,17,18]. Photosynthesis is the main physiological process that is affected by heat. High temperatures modify the microscopic ultrastructure of chloroplasts, altering membrane fluidity and increasing thylakoid leakiness [19]. Heat damage in chloroplasts occurs principally on photosystem II (PSII) [20,21,22,23], the oxygen-evolving complex (OEC) [24], the ATP-generating system, and the cytochrome b6f complex [25,26]. In addition, HS also affects the activity of RUBISCO activase [27,28], the metabolism of carbon assimilation, and chloroplast biosynthesis, leading to leaf senescence, which is a clear symptom of heat injury [29,30,31,32]. At the cellular level, HS increases the production of reactive oxygen species (ROS), which directly or indirectly may cause many injuries such as protein denaturation, the inhibition of protein synthesis, the loss of membrane and cytoskeleton integrity, and the inactivation of enzymes [33,34].

As sessile organisms, plants have developed sophisticated signaling networks to sense HS and react to the harmful effects of high temperatures [34,35,36,37,38]. Since photosynthetic apparatus is the primary target of heat damage, chloroplasts can act as sensors of HS [26,38], activating retrograde signaling that induces changes in the expression of nuclear-encoded genes for the metabolic and molecular reprogramming required for stress adaptation [39,40,41]. One of the most well-studied mechanisms of the heat stress response (HSR) is the intricate network of heat shock factors (HSFs) and heat shock proteins (HSPs). HSPs are chaperone proteins that are able to repair aggregated, denatured, or misfolded proteins, allowing plants to maintain their delicate metabolic processes during heat and other stress [42].Through their association with the heat shock element (HSE), in the promoter of *HSPs* and other heat-responsive genes, HSFs alter gene expression to obtain adjustments in the molecular pathways involved in enabling plants to survive under high temperatures [43].

In recent years, many data have highlighted a significant role of ROS as signaling molecules in the HSR [44,45]; moreover, several genetic and biochemical studies have indicated that complex interactions take place between HSFs/HSPs, ROS, and the heat acclimatation response [46]. This review aims to provide and discuss data on the role of ROS and the redox-related signaling pathways that are involved in the acquisition of thermotolerance.

## 2. Production of Reactive Oxygen Species in Heat Stress Conditions

High temperature, like many environmental stresses, leads to the overproduction of ROS, the reactive forms of molecular oxygen (O_2_), including singlet oxygen (^1^O_2_), superoxide anion radical (O_2_^•−^), hydrogen peroxide (H_2_O_2_), and hydroxyl radical (HO^•^). Under environmental stress conditions, ROS accumulation results in oxidative damage to biomolecules, such as pigments, proteins, lipids, carbohydrates, and DNA, leading to cellular injuries and cell death. However, a fine balance between production and scavenging, controlling ROS levels, allows these molecules to act as molecular signals that induce changes in cellular metabolism, leading to adaptative responses or triggering programmed cell death (PCD) [47,48].

Under abiotic stress, ROS production occurs in different cellular compartments, such as chloroplasts, mitochondria, peroxisomes, cell wall, apoplast, and plasma membrane. During stress conditions, ROS production occurring in the photosynthetic and respiratory electron transport chains performs a regulatory function in alleviating over-reduction [49]. Moreover, the redox state of chloroplasts, peroxisomes, and mitochondria is connected by photorespiration, which is affected by several environmental stresses [50]. ROS accumulation in different organelles strictly depends on the plant tissues. In organs with low photosynthetic metabolism, such as fruits and flowers, ROS are principally formed in the mitochondria, peroxisomes, and apoplast [51], while in photosynthetic tissues, chloroplasts are the prime source of ROS [52]. Chloroplasts act as the principal sensors of environmental stresses, including heat [53]. HS in chloroplasts reduces photosynthetic efficiency, deactivating the reaction center of PSII and inhibiting electron flow to the plastoquinone pool and the induction of non-photochemical quenching [54]. Damage to the photosynthetic components can lead to excess excitation energy, resulting in the formation of a chlorophyll triplet state that reacts with molecular oxygen, forming ^1^O_2_ [55]. ^1^O_2_ formation at high temperatures is also associated with heat-induced lipid peroxidation in the thylakoid membrane and damage to PSII [56]. In the unicellular green alga, *Chlamydomonas reinhardtii*, which is exposed to 40 °C, it has been suggested that ^1^O_2_ formed by lipid peroxidation is initiated by an enzymatic reaction that is catalyzed by lipoxygenase [57]. Additionally, ^1^O_2_ can be produced by the direct decomposition of tetraoxide via the Russell reaction [57].

In chloroplasts, O_2_^•−^ is formed by three processes: (i) in PSII, associated with incomplete water oxidation, (ii) in the electron transport chain, via plastosemiquinone interaction with O_2,_ and (iii) in Photosystem I (PSI), due to the oxidation of ferredoxin that reduces O_2_ to O_2_^•−^ and that takes place when the Calvin–Benson cycle does not operate properly and lowers NADP^+^ availability [50,53]. O_2_^•−^ is disproportionate to H_2_O_2_ and O_2_ in a reaction catalyzed by superoxide dismutase (SOD) [58]. Moreover, H_2_O_2_ can also be produced in PSII when HS causes the cleavage and degradation of the D1 protein with the consequent release of PsbO, PsbP, and PsbQ [23,56]. These three proteins, which together with the inorganic cluster of Mn_4_O_5_Ca form the OEC, are located on the inner surface of the thylakoid membrane, in close association with PSII, and are involved in its thermal stability [59]. Indeed, the release of PsbO, PsbP, and PsbQ proteins leads to improper H_2_O accessibility to the water-splitting manganese complex and the consequent formation of H_2_O_2_. On the PSII electron donor site, H_2_O_2_, formed by incomplete water oxidation, can produce the more toxic HO^•^, through the Fenton reaction [60].

Under HS, the disturbances of respiratory metabolisms in the mitochondria trigger impairments of the electron transport chain (ETC), resulting in an increase in ROS levels. In tobacco cells, an impaired mitochondrial metabolism is responsible for the oxidative burst arising during heat-induced PCD [61,62]. O_2_^•−^, which is formed when a single electron leaks to O_2_ in ETC components, can act as a substrate for the generation of H_2_O_2_ by matrix-localized manganese SOD (MnSOD) [63]. HS promotes Ca^2+^ increase in mitochondria, which is accompanied by hyperpolarization of the inner mitochondrial membrane, leading to an increase in ROS generation [64]. HS increases membrane fluidity, impairing cytochrome c oxidase (COX), resulting in an over-reduction in the ETC that culminates in ROS production [65,66]. The maintenance of COX integrity is favored by the mitochondrial HSP70-1; consistently, *mtHSC70-1* knockout lines show decreased COX activity and an increase in ROS production [67]. Additionally, during HS, an induction of *COX1*, *COX2*, and *COX3* genes has been reported in Arabidopsis [7], highlighting the fact that plants try to counteract HS injury to mitochondria by preventing imbalances in the ETC. Moreover, in plants, the presence of alternative oxidase (AOX), which catalyzes the oxidation of ubiquinol and the reduction of O_2_ to H_2_O, helps to maintain electron flow, preventing the over-reduction in the ETC and O_2_^•−^ formation [68]. The increase in the AOX protein after exposure to high temperatures confirms the participation of this enzyme in HSR [69].

During HS, ROS generation also comes from the activity of the plasma membrane NADPH-oxidase, which transfers an electron from intracellular NADPH to O_2_ in the apoplast, generating O_2_^•−^, which is then converted into H_2_O_2_ by SOD [70,71]. NADPH-oxidase is controlled by phosphorylation and by the direct binding of calcium. Thus, cytosolic calcium influx, occurring as a result of heat perception, activates the enzyme and leads to ROS accumulation [72]. In Arabidopsis, NADPH-oxidase is encoded by ten genes (respiratory burst oxidase homologues—RBOHs), which are involved in several biological processes, including biotic and abiotic stress responses. *AtrbohB* and *AtrbohD* play a key role in HSR, as suggested by the heat-sensitive phenotype observed in *atrbohB* and *atrbohD* Arabidopsis mutants [73]. In rice, six NADPH-oxidase genes increase their expression in response to HS [74]. NADPH-oxidase has a crucial function in thermotolerance acquisition, enhancing the expression of heat-induced genes and controlling the H_2_O_2_ level by regulating antioxidant enzymes [75].

## 3. Role of Antioxidants in Heat Stress Response

The role of ROS as toxic or signaling molecules depends on a delicate equilibrium between the production and scavenging of these reactive species in different cell compartments [46,47]. Antioxidants are the first line of defense against oxidative damage and their activity is critical to maintaining an optimal redox environment. The antioxidant machinery includes non-enzymatic components, such as ascorbate (ASC), glutathione (GSH), carotenoids and tocopherols, as well as enzymatic components such as ascorbate peroxidase (APX), catalase (CAT), SOD, peroxidases (PODs), glutathione peroxidase (GPX), glutathione S-transferase, and the enzymes involved in the reduction of the oxidized forms of ASC and GSH in the ASC–GSH cycle, namely, monodehydroascorbate reductase, dehydroascorbate reductase, and glutathione reductase (GR) [76].

A primary strategy used to Iimpart thermotolerance in plants involves enhancing the antioxidant machinery. In wheat, HS is better tolerated by plants that are subjected to heat priming. The improvement of thermotolerance is related to the enhanced activity of antioxidant enzymes, particularly to the upregulation of chloroplastic Cu/Zn-SOD and mitochondrial GR and POD [77,78]. Consistently, in Chinese Spring, an HS-sensitive wheat variety, the significant decrease in SOD, CAT, POD, and GPX activities, occurring after exposure to high temperature, causes ROS accumulation and increased levels of oxidized lipids [79]. In apple trees, HS (48 °C for 6 h)—despite the increases in antioxidant activity—causes a rise in H_2_O_2_, O_2_^•−^ and lipid peroxidation, and a decrease in chlorophyll content. The overexpression of *MdATG18a*, encoding a key protein involved in autophagy, removes damaged chloroplasts and enhances antioxidant activity, thus improving the expression of heat-related genes. In transgenic plants, the reduction of ROS accumulation confers thermotolerance [80].

Many treatments that confer thermotolerance activate antioxidant machinery. In potato seedlings, a pre-treatment with sucrose, before exposure to HS, enhances SOD, CAT, APX, and POD activity, reducing ROS accumulation and resulting in increased thermotolerance [81]. Similar results have been obtained in potato plants treated with exogenous silicon, which mitigates heat-induced oxidative stress by increasing the expression of antioxidant enzymes [82]. In strawberries, the enhanced transcription of biosynthetic enzymes of ASC and GSH and antioxidant enzymes (APX, CAT, SOD, and GR), occurring in plants pre-treated with sodium hydrosulfide before HS, leads to a higher stress tolerance [83]. Furthermore, tomato thermotolerance due to the exogenous application of rosmarinic acid, a plant-derived phenolic compound, is linked to the increased transcript abundance and activity of ASC–GSH cycle enzymes, which modulate the ASC and GSH redox state [84].

The behavior of antioxidant machinery to counteract and/or mitigate heat injury depends on plant species, genotypes, phenological stage, and stress severity [85,86,87,88,89,90]. Moreover, different isoforms of antioxidant enzymes can show different performance in HSR; therefore, changes in the antioxidant systems in response to HS denote a complex scenario with a number of controversial points.

Among the antioxidant enzymes, CAT is one of the main scavengers of H_2_O_2_. Arabidopsis plants possess three CAT isoenzymes differing in their subcellular localization and exhibiting different tissue specificity. Analysis of *cat* mutants has suggested that CAT2 has a greater effect in removing H_2_O_2_ compared to CAT1 and CAT3, which are mainly “backup” or stress-specific enzymes [91]. Studies on Arabidopsis mutants lacking the three CATs have revealed that only *cat2*, but not *cat1* and *cat3*, are hypersensitive to moderate long-term HS (35 °C for 5 days), but the same mutants do not differ from the wild type (wt) in response to short-term HS (42 °C for 40 min). However, the CAT2-overexpressing lines do not show improved thermotolerance, suggesting that CAT2 is necessary but insufficient to enhance long-term HS response [92]. In *Brassica napus* seedlings, the total CAT activity decreases after 24 and 48 h of HS at 38°; nevertheless, after selenium supply, which confers HS-tolerance, the increase in CAT activity, along with the subsequent reduction in oxidative stress, indicate a role for CAT in H_2_O_2_ detoxification, which is responsible for thermotolerance [93].

APX is a plant antioxidant enzyme that uses ascorbate as an electron donor and plays a key role in the scavenging of H_2_O_2_. In Arabidopsis, there are different isoenzymes classified based on their subcellular localization and that target cytosol (i.e., APX1 and APX2), mitochondria (mitAPX), chloroplastic stroma (sAPX), and thylakoid membranes (tAPX) [94]. Both cytosolic isoforms possess a heat shock element (HSE) in the promoters and are induced during HSR [95,96,97,98]. Arabidopsis *apx2* mutants are more sensitive to HS at the seedling stage, showing a lower survival rate than the wt, but showing higher thermotolerance at the reproductive stage, producing more seeds under prolonged heat stress. This finding supports the idea that the lack of APX2 induces a signal suppressing ROS accumulation during stress, through the activation of different redundant ROS scavenging systems or the suppression of ROS production and that this signal may be stage- or tissue-specific [99]. The phosphorylation of APX2 by the calcium-dependent protein kinase CPK28 is needed to enhance thermotolerance; indeed, *cpk28* mutants show increased HS-induced ROS accumulation and protein oxidation together with the reduced activity of APX [100]. The lack of APX1 in Arabidopsis causes stunted growth and enhanced sensitivity to oxidative stress [101]; moreover, APX1 seems to be necessary to increase tolerance to HS combined with drought [102,103]. Interestingly, APX1 subjected to heat treatment shows conformational changes, passing from dimeric to high-molecular-weight structures, losing peroxidase activity and acquiring chaperone activity, which can confer thermotolerance [104].

Under HS, the expression profile of APX1 and APX2 is different; in Arabidopsis, APX1 mRNA is already present in unstressed conditions and shows a modest increase under moderate HS, whereas APX2 mRNA is not detected in control conditions and has a fast, transient (1–2 h), and strong induction after HS [95,96]. Similarly, in independent studies, the maximal expression of APX1 and APX2 has been observed after 24 h and 15 min of HS, respectively [98,105]. These data suggest a similar but not superimposable role of these two isoforms.

The involvement of APX in HSR has also been widely studied in tobacco BY-2 cells. In these cells, short-term HS at 35 °C or 55 °C causes an increase or a decrease in APX activity, respectively [106]. The increase in APX activity under moderate HS is only imputable to the cytosolic isoenzymes, whereas exposure at 55 °C decreases all of the APX isoenzymes [107]. In this context, the inhibition of APX activity causes ROS accumulation and participates in the induction of PCD [61]. The reduction in cytosolic APX activity under PCD conditions is a precocious event, due to nitrosylation, ubiquitination, and the consequent proteasome-dependent degradation of the protein [108]. In tobacco BY-2 cells, cytosolic APX behaves differently depending on the duration of the moderate HS: activity and expression increase in the first 3 days of HS. However, when HS is protracted, a drop in enzyme levels is responsible for the rise in oxidative damage, leading to cell death [109].

Inconsistent behavior of isoenzyme regulation in the HSR has also been reported for SODs. In tomato plants, heat acclimation before HS favors an increase in the total SOD activity, but while Fe-SODs and Mn-SODs are induced, Cu/Zn-SODs decrease. The increase in Fe-SOD activity seems to be strictly related to conditions where the photosynthetic functions are unaffected by heat, indicating that Fe-SODs could participate either in reducing the spread of ROS and also protecting the thylakoid membrane and PSII reaction center from oxidative damage [110,111]. More recently, it has been reported that the decrease in Cu/Zn-SOD activity, with concomitant ROS accumulation, enhances thermotolerance in Arabidopsis [112]; similarly, a decrease in Cu/ZnSOD and Fe-SOD mRNA levels has been observed in Arabidopsis subject to 45 °C for 2 h [113]. This scenario suggests that the activity of these antioxidant enzymes in chloroplasts may be downregulated by HS, thus exacerbating ROS accumulation, in order to obtain cellular environment oxidation that functions as a signal to activate stress-responsive systems [113].

In tomato, an increase in APX and SOD activities, but not in gene expression, occurs after HS. The increase in the activity of these antioxidant enzymes is due to the induction of the HSP40, LeCDJ1, which acts as a chaperone that protects APX and SOD protein conformation, increasing their enzymatic activity, alleviating PSII damage, and conferring heat tolerance [114,115]. More recently, it has been reported that HSP40 interacts with SISNAT, an enzyme located in the chloroplast that participates in the biosynthesis of the ROS scavenger melatonin. SISNAT-overexpressing lines subjected to HS show decreased ROS levels, increased RUBISCO protection, and an upregulation of HSFs and HSPs [116]. Consistently, treatment with exogenous melatonin before HS increases thermotolerance, as a consequence of the reduction in oxidative stress [117,118].

HS tolerance in plants is also associated with non-enzymatic antioxidants. In mung bean plants, HS at 42 °C leads to a decrease in the ASC content and GSH/GSSG ratio, with a concomitant increase in oxidative markers; however, the supply of exogenous GSH induces tolerance to high temperatures by improving the antioxidant systems [119]. Similar results have been reported in tomato, where the decrease in GSH/GSSG ratio, which is attributed to the impairment of GSH recycling, supports the idea that the maintenance of GSSG under stress conditions is an indicator of high ROS accumulation [120,121].

## 4. Reactive Oxygen Species as Signaling Molecules in the Heat Stress Response

**Figure 1:**
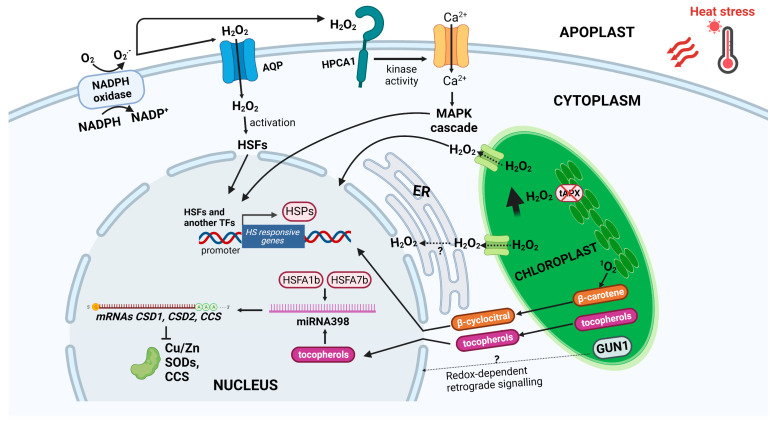
Mechanisms of ROS accumulation and signaling in heat stress response. After heat stress, production, and accumulation of reactive oxygen species (ROS) occurs in both chloroplasts and apoplast. ^1^O_2_ accumulation in the chloroplast, oxidizing β-carotene, produces β-cyclocitral, which acts as a retrograde signal from chloroplast to nucleus, activating heat stress-responsive (HSR) genes. In chloroplasts, damage to photosynthetic apparatus, as well as inhibition of tAPX, lead to accumulation of H_2_O_2_, which moves to the nucleus through aquaporins, and/or ER, thus inducing HSR genes. The chloroplast-localized GUN1, by the redox-dependent plastid to nucleus communication, is involved in cell environment oxidation. ROS increase is also due to downregulation of *CSD1*, *CSD2*, and *CCS* by miR398, whose accumulation is dependent on HSFA1b and HSFA7b. Tocopherols produced in the chloroplast participate in miR398 induction. H_2_O_2_ produced in the apoplast by NADPH oxidase can diffuse in the cell via aquaporins, and/or sensed by membrane receptor HPCA1, which stimulates a calcium influx, triggering the MAP kinases cascade and transcription of HSPs. Abbreviations: ^1^O_2,_ singlet oxygen; tAPX, thylakoidal ascorbate peroxidase; H_2_O_2_, hydrogen peroxide; ER; endoplasmic reticulum; GUN1, genomes uncoupled1; *CSD*, Cu/Zn superoxide dismutase; *CCS*, Cu chaperone for superoxide dismutase; HSF, heat shock factor; HPCA1, HYDROGEN-PEROXIDE-INDUCED CALCIUM INCREASES1; MAP; mitogen activated protein; and HSP, heat shock protein. Created with BioRender.com; accessed on 3 February 2023.

The heat-induced ROS accumulation is frequently considered in terms of oxidative stress, cell damage, and death. However, ROS can act as signaling molecules crucial for the acquisition of thermotolerance (Figure 1) [4,12,68,79,114,115,116,122,123]. Indeed, the amount of H_2_O_2_ increases after the exposure to high temperatures, causing HSFs activation and the accumulation of HSPs [124]. Consistently, in HS-exposed plants, inhibitors of H_2_O_2_ synthesis and ROS scavengers can prevent HSP expression [125]. Studies on mutants of ROS biosynthesis corroborate the idea of ROS requirements for achieving thermotolerance. Mutations in *AtrbohB* and *AtrbohD*, which participate in H_2_O_2_ production in the HSR, lead to an increased heat sensitivity [73]. In addition to not accumulating H_2_O_2_, *atrbohB*, *atrbohD*, and *atrbohB/D* double mutants fail to induce the expression of HSP17.7 and HSP21 [126]. H_2_O_2_ formed in the apoplast can be promptly sensed by membrane receptor kinases such as HPCA1, which causes a calcium influx that activates MAP kinases and additional signaling pathways [127].

The downregulation of ROS scavenger enzymes also contributes to ROS accumulation and the activation of HSFs, giving a positive feedback loop regulating thermotolerance. During HS, HSFA1b and HSFA7b induce miR398, which downregulates *CSD1*, *CSD2*, and *CCS* genes, encoding cytosolic and chloroplast Cu/Zn SODs and a copper chaperone that delivers Cu for both SODs, respectively. Therefore, ROS accumulate and induce other HSF genes, promoting heat stress tolerance. Consistently *csd1*, *csd2*, and *ccd* mutants and plants over-expressing miRNA398 are more heat tolerant [112]. The lipid soluble antioxidant tocopherols, synthesized in chloroplast during HS, positively regulate the biogenesis of miRNAs, and this regulation involves the heat-induced accumulation of miRNA398. In the biosynthetic tocopherol mutant *vte2-1*, the induction of miR398 and suppression of *CSD2* by heat are abolished; additionally, knocking down *CSD2* in *vte2-1* restores heat tolerance, supporting the evidence that SODs inhibition and ROS accumulation increase thermotolerance (Figure 1) [128].

Chloroplasts activate retrograde signaling to communicate environmental stress and induce changes in the expression of nuclear-encoded genes, which bring metabolic and molecular reprogramming for stress adaptation. Chloroplasts have been shown to play an important role in heat-induced ROS accumulation and the subsequent expression of nuclear heat-responsive genes [129]. ROS produced in chloroplasts can work as plastid signals to activate the expression of genes coding for antioxidant enzymes and to fine-tune the stress-responsive apparatus for a more effective adaptation to stresses [40].

Among ROS, ^1^O_2_ and H_2_O_2_ impact nuclear gene expression extensively. Singlet oxygen can function as a retrograde signal to activate nuclear gene expression. Given the very short half-life of ^1^O_2_ (200 ns) and its high reactivity, it can be located exclusively inside chloroplasts. Thus, more stable second messengers derived from ^1^O_2_ within the plastids are assumed to activate signaling pathways in controlling the expression of nuclear genes [40,130,131,132]. The ^1^O_2_-induced pathway involves the accumulation of β-cyclocitral, an oxidation product of β-carotene. β-cyclocitral could directly travel to the nucleus and activate ^1^O_2_-dependent retrograde signaling; it is positively correlated with heat tolerance because it is directly involved in the induction of responsive genes, such as genes responding to oxidative stress, HSE-binding proteins, and various defense genes (Figure 1) [125,126,127]. H_2_O_2_ derived from chloroplasts also acts as a specific signal for regulating plant responses to stress. In Arabidopsis, the t-APX silencing, increasing H_2_O_2_ levels in chloroplasts, affects the expression of a large set of genes associated with stress [133]. GUN1, a key player of plastid-to-nucleus retrograde signaling [134,135,136], has been proposed to be involved in ROS accumulation occurring in response to HS. Indeed, unlike wt plants, *gun1* mutants do not accumulate O_2_^−^ and H_2_O_2_, failing to induce the transient oxidative burst needed for HSR and resulting in more HS sensitivity [113] (Figure 1).

The direct transfer of H_2_O_2_ from chloroplasts to the nucleus has been proposed as a signaling way to induce nuclear heat-associated gene expression and the close association between the two compartments may provide the specificity needed for retrograde signal transduction. So that H_2_O_2_ passes from the chloroplast to the nucleus, it must cross the chloroplast double envelope, the perinuclear space, and the inner nuclear membrane; this movement probably occurs by diffusion via water channels [137]. Stromules may also promote chloroplasts-to-nucleus contacts, transferring H_2_O_2_ to the nucleus [138]. The close association between the endoplasmic reticulum (ER) with chloroplasts and nucleus has led to suppose that the ER may play a key role in mediating H_2_O_2_ retrograde signaling in a double manner: amplifying the signal, generating more H_2_O_2_ by a luminal ER oxidase, or attenuating it by activating antioxidant responses such as the ER glutathione peroxidase [139] (Figure 1).

## 5. Heat Stress-Dependent Crosstalk between Signaling Networks of Reactive Oxygen Species and Hormones

**Figure 2:**
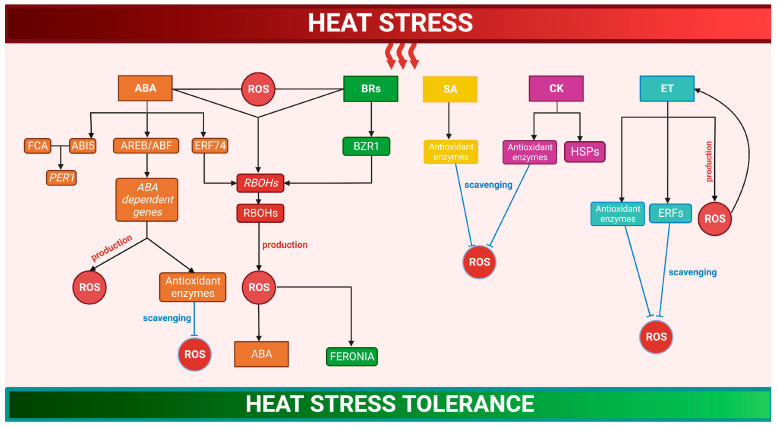
Crosstalk between signaling networks of reactive oxygen species and hormones in heat stress response. Under HS, ABA activates AREB/ABF transcription factors controlling ABA-dependent gene expression, stimulating ROS production, or improving antioxidant machinery. ABA-induced ERF74 also increases ROS production by RBOHs induction. The interaction of FCA with ABI5 increases thermotolerance through the transcriptional regulation of PER1. BRs, by activating RBOHs, stimulate a transient H_2_O_2_ accumulation, which increases ABA levels resulting in additional rises in H_2_O_2_. In BR-mediated heat tolerance, the regulator BZR1 improves the production of ROS, which regulates the receptor-like kinase FERONIA. SA, CK, and ET enhance antioxidant systems, increasing ROS scavenging. In addition, CK stimulate HSPs. ROS can act in concert with ET in a self-intensifying feedback loop where ET stimulates H_2_O_2_ accumulation, which in turn improves ET production. ERFs contribute to lower ROS accumulation, balancing redox homeostasis. (See more details in the text). Abbreviations: ABA, abscisic acid; AREB/ABF, ABRE-binding/ABRE-binding factors; ERF74, ethylene response factor 74; RBOH, respiratory burst oxidase homolog; FCA, flowering control locus A; ABI5, ABA-INSENSITIVE 5; PER1, 1-Cys peroxiredoxin; BRs, brassinosteroids; BZR1, brassinosteroid-regulated transcription factor 1; SA, salicylic acid; CK, cytokine; ET, ethylene; and ERF, ethylene response factors. Created with BioRender.com.; accessed on 20 February 2023.

During HS, ROS signaling is also modulated by a wide interaction with hormone signaling [140,141]. Indeed, ROS and hormones that are apart to cooperate as single molecules are also connected by crucial signaling factors, such as calcium, cyclic nucleotides, MAPKs, G-proteins, and various transcription factors or regulator molecules. This delicate communication between ROS and hormones signaling organizes a suitable response that permits the plant to overcome the stress condition (Figure 2) [35,142,143,144,145].

Hormones induced by HS can modify ROS amounts, activating RBOHs or modifying redox signaling, or ROS can act upstream the hormones. Moreover, ROS can play a role in mediating the interaction between different hormones, thus improving HS tolerance. These interactions lead to an amplification loop, controlling the gene expression and HSR [142,143,146,147,148].

After HS, the increase in abscisic acid (ABA) levels activates the transcription factors, such as AREB/ABFs, controlling the ABA-dependent gene expression that stimulates ROS production or improves antioxidant machinery [35,149,150,151,152]. For instance, the interaction of flowering control locus A, a plant-specific RNA-binding protein of the autonomous flowering pathway, with ABA-INSENSITIVE 5 increases thermotolerance through the transcriptional regulation of the PER1 gene encoding 1Cys-PRX [149]. The ABA-induced ERF74 induces RBOHD transcription, increasing ROS production, and enhancing HS tolerance [153] (Figure 2). The transcriptional co-activator MBF1c, as well as transcription factors of DREB2A and AP2-EREBPs families, induced by both ROS and ABA, play key roles in thermotolerance acquisition [103,154,155]. In the interaction between the regulative pathways of ROS, ABA, and auxin, the ROS-scavenger enzyme APX6 is involved in the HS protection of seeds during germination [156].

In the HSR of tomato plants, a crosstalk between ABA and brassinosteroids (BRs) mediated by ROS occurs. BRs, by activating RBOHs, stimulate a transient H_2_O_2_ accumulation, which increases the ABA levels and results in additional rises in H_2_O_2_ and enhanced HS tolerance [146]. In BR-mediated tolerance to HS, the regulator BZR1 improves the production of apoplastic H_2_O_2_ that regulates the receptor-like kinase FERONIA, resulting in HS tolerance [157] (Figure 2).

During heat stress, salicylic acid (SA) also increases and protects PSII from the high ROS levels [158,159]. The Arabidopsis mutants *sid2*, which are deficient in SA signaling, accumulate elevated ROS levels and reveal increased HS sensitivity, showing that SA has a crucial role in thermotolerance [154,155,160]. In tomatoes and barley, SA enhances antioxidant systems, increasing ROS scavenging and heat tolerance [161,162]. Moreover, treatment with SA improves the activity of antioxidant enzymes and by controlling ROS level, it relieves the heat-dependent decline in pollen viability and floret fertility [163,164] (Figure 2).

Ethylene (ET) production during HS has been related to the beginning of leaf senescence and reduction in pollen growth and germination, spike fertility, and grain weight, which are survival strategies to preserve the optimal progeny capacity under high temperatures [165]. Under HS, ROS can act in concert with ET in a self-intensifying feedback loop where ET stimulates H_2_O_2_ accumulation, which in turn improves ET production, initiating leaf senescence or chlorosis [142,166]. ET can additionally control ROS metabolism by altering antioxidant enzymes [167]. Rice seedlings treated with the ET precursor, 1-aminocyclopropane-1-caroboxylic, recover the activities of CAT, APX, and POD and show a decreased H_2_O_2_ accumulation and cell damage under HS [168]. Moreover, ET-induced ERFs are involved in redox regulation, while oxidative stress induces ERFs [169]. In tobacco, ERF3 contributes to lower ROS accumulation, enhancing abiotic stress tolerance [170], while in Arabidopsis, ERF6 controls ROS signaling during HS. These data suggest that ERFs control HS tolerance by modulating ROS homeostasis [171] (Figure 2).

Heat stress reduces the endogenous levels of cytokinins (CKs) [172]. However, the treatment with CKs usually improves HS tolerance by the increase in antioxidant enzymes and HSPs [173]; consistently, the inhibition of CKs degradation improves HS resistance, principally by the enhancement of antioxidant enzymes [173,174,175] (Figure 2).

## 6. Heat Stress-Dependent Oxidation of Cellular Environment

The increase in temperature provokes an enhanced ROS production and accumulation that temporarily oxidizes the cellular environment, triggering downstream signaling cascades. Because of their high reactivity, when ROS escape antioxidant-mediated scavenging, they react with biomolecules causing lipid peroxidation, protein oxidation, and other protein modification, which often might serve as signaling factors that trigger the effective defense response [176]. Thus, not only ROS accumulation, but also the HS-related redox changes are closely associated with the HSR that supports thermotolerance. Indeed, the inhibited expression of scavenger enzymes leads to an increase in the oxidative power required to activate the HSFs in the initial steps of HSR [46]. Consistently, miRNA398, which downregulates the CSD1, CSD2, and CCS genes, achieves its highest expression within 2 h from HS; moreover, heat improves tolerance to subsequent high temperature stress [77,177]. In Arabidopsis, a transient oxidative burst, occurring immediately after HS and which is due to a reduction in APX and CAT activity, causes a transient oxidation of the cellular environment, which helps trigger a signaling cascade that protects plants from HS. Accordingly, after 3 h of physiological recovery from HS, both non-enzymatic and enzymatic antioxidants significantly increase, lowering ROS accumulation and preventing oxidative damage. On the other hand, *gun1* mutants that fail to increase ROS levels promptly exhibit impaired HSR and accumulate ROS and oxidative damage during physiological recovery at the growth temperature. Thus, GUN1 seems to be required to oxidize the cellular environment, participating in the acquisition of basal thermotolerance through the redox-dependent plastid-to-nucleus communication [113].

A recent study on HS response, performed using the redox-sensitive green fluorescent protein, roGFP2, clearly demonstrated that HS, by triggering large shifts in the glutathione redox potentials of cells, causes a huge rise in the extent of oxidation of both cytosol and nuclear compartments [105]. In this context, the analysis of transcript profiles indicates a very specific redox-processing response to HS, since APX2 was the only increased antioxidant transcript; moreover, the enhanced expression of ZAT12 in the seedlings after HS suggests altered oxidative stress signaling [105,178].

Cellular environment oxidation permits redox-dependent changes to proteins that include conformational changes of proteins involved in HSR, as well as modifications to transcription factors or associated proteins that can also trigger nuclear translocation [179]. Cysteine (Cys) residues of proteins may act as sensors of redox changes triggering different post-translation modifications, which in turn modulate redox-dependent pathways until a plant’s response to environmental insults is achieved [180,181]. Thiol residues could be deprotonated into a thiolate residue (R-S-), which leads to successive oxidations to sulfenic, sulfinic, and sulfonic acids [182]. Thiol groups can also form a disulfide bridge (S-S) or react with reactive nitrogen species (RNS), resulting in S-nitrosylation. Depending on their nature, most of these thiol modifications can be reversed by dedicated thiol reduction systems (thioredoxin-Trx, glutaredoxins-GRX, and S-nitrosoglutathione reductase GSNOR).

Exposure to high temperatures usually leads to an increase in RNS, which are essential for the acclimatization mechanism [183,184]. It has been demonstrated that the exogenous application of nitric oxide (NO) to plants subjected to HS promotes the activation of enzymatic and non-enzymatic defense systems to oxidative stress [122,185,186]. In contrast, treatment with NO scavengers eliminates the beneficial effects caused by NO, corroborating the idea that NO is involved in tolerance to high temperatures [185,187]. S-nitrosoglutathione reductase, GSNOR1, controls the turnover of GSNO under stress conditions [188,189]. In the *Arabidopsis thaliana* thermotolerance-defective mutant, *hot5* (sensitive to hot temperature 5), with HOT5 encoding GSNOR1, the loss of enzymatic activity increases s-nitrosothiols and leads to a high heat sensitivity [190]. Thus, the absence of GSNOR1 activity undeniably affects NO/GSNO levels, which might correlate with heat sensitivity and thermotolerance [190,191].

## 7. Redox-Dependent Changes of Proteins Involved in the Heat Stress Response

ROS work efficiently in cell signaling through chemical reactions with specific target protein residues that lead to protein modifications. The primary targets of ROS are proteins containing amino acids with Cys residues. The redox changes that involve thiol-disulfide switches bring about various effects, such as the induction of protein conformational changes, the alteration of protein–protein interactions, and changes in protein subcellular localization [192].

In mild HS, any imbalance in the redox state of the chloroplast may lead to the inhibition of a specific redox-sensitive kinase (STN7), a transmembrane protein with catalytic domains containing two conserved Cys residues. STN7 phosphorylates the light-harvesting complex II (LHCII), which migrates to PSI and shifts the excitation energy to PSI. Thus, the redox-dependent STN7 inhibition, which alters LHCII phosphorylation, does not permit the formation of the PSI-LHCII complex with a consequent alteration in the thylakoid ultrastructure and a strong depletion in the photosynthetic rates [193]. Interestingly, one of the four phosphorylation sites in the C-terminal region of STN7 is phosphorylated by CK2, which is a chloroplast nuclear casein kinase regulated by the redox signal [194].

Under heat stress, many proteins undergo redox-dependent conformational changes that are associated with the acquisition of a new function (Table 1).

An interesting example showing how changes in the protein oxidation state lead to a switch of function during HS is represented by the Arabidopsis h-type thioredoxin (Trx), AtTrx-h3. This protein forms several protein structures varying from low and oligomeric to high molecular weight (HMW) complexes, performing the function of disulfide reductase in the low molecular weight (LMW), but acting as a molecular chaperone in the HMW complexes. The structures of AtTrx-h3 are regulated both by HS and the redox state. Two active Cys residues in AtTrx-h3 are necessary for disulfide reductase activity, but not for the chaperone function. The reduction of AtTrx changes the HMW structures into LMW, while oxidation permits the formation of the HMW structure. It has been demonstrated that AtTrx-h3 confers HS tolerance in Arabidopsis, mainly in the oxidized state, through its chaperone function [195].

Another redox protein revealing an analogous regulation mode of AtTrx-h3 is the C-type NADPH-dependent Trx reductase (NTRC), a member of the plant-specific NADPH-dependent Trx reductase family, which comprises an N-terminal Trx reductase domain and a C-terminal Trx domain. This protein works as an efficient electron donor to 2-Cys peroxiredoxins [200,201]. The structure of NTRC shows different oligomeric conformations in various plant species [202,203,204], acting as a disulfide reductase and a foldase and holdase chaperone, depending on the protein structures. The LMW structures display greater disulfide reductase and foldase chaperone activities, while HMW ones have stronger activity as a holdase chaperone. Heat stress mediates the redox-dependent oligomeric changes of NTRC, converting LMW proteins into HMW complexes and progressively enhancing the holdase chaperone activity, which confers higher thermotolerance [196].

The 2-Cys peroxiredoxins (Prx) belong to the Prxs family, which are peroxidases that reduce H_2_O_2_, peroxynitrite, and alkyl hydroperoxides [197,205,206]. The overexpression of 2-Cys Prx in *Festuca arundinacea*, reducing oxidative damage under high temperature, confers thermotolerance [207]. The 2-Cys Prx is essential in protecting the cellular environment from oxidative stress, since under physiological conditions it forms LMW structures such as dimers through disulfide bonds and removes H_2_O_2_. However, an increase in ROS under HS leads to an overoxidation of the 2-Cys Prx, which, aggregating into HMW structures, loses its predominant function of antioxidant enzyme and gains the chaperone one, which is essential to mediate the proper folding of proteins in response to HS [197]. In this way, Prx can act as a sort of “dam” to H_2_O_2_ flow. In stress conditions, Prx inactivation may be a way to leave sufficient time for H_2_O_2_ to mediate stress signaling and to promote a variety of redox-dependent processes [208,209].

Redox-dependent conformational changes associated with different functions in response to HS also occur in the Arabidopsis glutaredoxin GRXS17: this member of the thiol reductase families in Arabidopsis is a nucleocytosolic monothiol GRX, containing an N-terminal thioredoxin domain and three conserved monocysteinic active sites that coordinate three iron–sulfur (Fe-S) clusters in a glutathione-dependent manner. GRXS17 is involved in the maturation of cytosolic and nuclear Fe–S proteins. However, GRXS17 also shows foldase and redox-dependent holdase activities. After HS, this protein loses its Fe–S clusters, forming disulfide bonds between Cys in the Trx domains, thus leading to the formation of HMW structures, which shows holdase activity in vitro. Therefore, upon HS, GRXS17 shows a redox-dependent chaperone activity, interacting with different proteins and protecting them from heat damage [198]. Consistently, the expression of Arabidopsis GRXS17 in maize confers thermotolerance due to an enhanced chaperone activity. Under heat stress, the GRXS17-overexpressing maize lines show a reduced protein damage and higher thermotolerance in the reproductive stages with an increase in grain production when compared to non-transgenic lines [210].

The Arabidopsis non-expressor of pathogenesis-related protein 1 (AtNPR1), which plays a significant role in plant systemic acquired immune responses [211], has also been shown to have a redox-dependent chaperone activity that is required to protect plants from HS. As a result of its structural switch into oligomeric form, the recombinant AtNPR1 works as a protein chaperone. The structural changes and the chaperone activity are dependent on its redox state since dithiothreitol treatment dissociates its structure to a monomer and reduces its chaperone activity [199].

## 8. Redox-Dependent Changes of Proteins Impacting Gene Expression during Heat Stress Response

**Figure 3:**
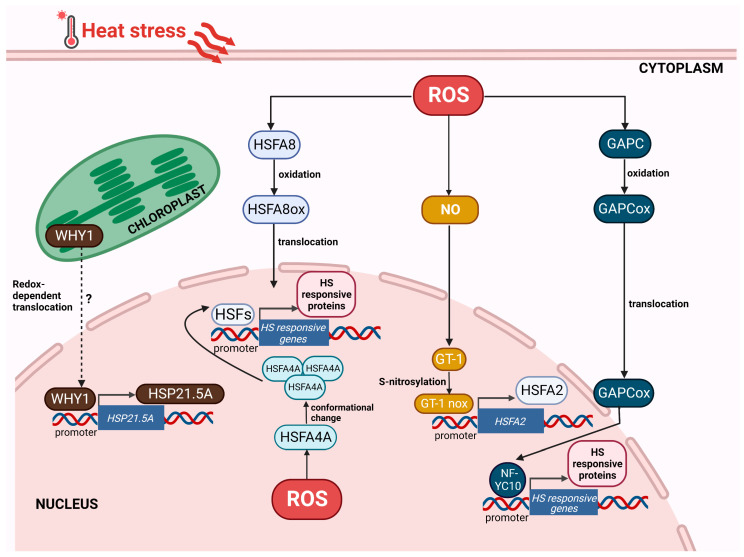
Redox-dependent changes of proteins impacting gene expression in response to heat stress. Heat stress, triggering oxidation of cellular environment, causes redox changes in proteins involved in the induction of heat stress response. The oxidation of HSFA8 and GAPC permits these proteins to move into the nucleus: HSFA8 directly and GAPC by the interaction with NF-YC10 promote the expression of heat stress-responsive (HSR) genes. S-nitrosylation of GT-1, triggered by ROS-dependent NO accumulation, induces the transcription of HsfA2. Nuclear redox oxidation is sensed by HSFA4A, which is stabilized and activated, inducing HSR genes. In the chloroplast, over-reduction in the components of the thylakoid electron transport chain might provoke the redox-dependent translocation of WHY1 in the nucleus, where the protein activates the transcription of HSP21.5A. Abbreviations: HSF, heat shock factor; GAPC, glyceraldehyde-3-phosphate dehydrogenase, cytosolic; NF-YC10, nuclear factor Y, subunit C10; and WHY1, Whirly1. Created with BioRender.com; accessed on 3 February 2023.

As a result of HS, the glutathione redox potentials of Arabidopsis cell nuclei are greatly changed, indicating nuclear oxidation [105]. Since transcript profiles of the control and heat-treated seedlings showed significant differences in HSPs, mitochondrial proteins, transcription factors, and other nuclear-localized components, the authors suggested that nuclear oxidation plays a key role in both the genetic and epigenetic control of HSR [105]. Nuclear oxidation can be responsible for the epigenome changes occurring in heat-induced priming [212], because many of the enzymes involved in histone and DNA methylation are subject to redox regulation [213,214]. Furthermore, nuclear oxidation may be responsible for the regulation of redox-sensitive transcription factors (Figure 3) [215].

It is known that HSFs sense the variation in temperature and in turn, modulate the expression of HSPs and other stress-associated genes [216]. In vitro stress treatments cause monomer-to-trimer transitions of HSFs, while the presence of the reducing agent dithiothreitol reverses this action. HSFs have been proposed to participate in peroxide sensing, as H_2_O_2_ can stabilize HSF trimers by reversible oxidation of Cys residues and by forming Cys–Cys bonds [217,218]. HSFA1a, the master regulator of HSR [219,220], contains one Cys residue located at the N-terminal portion of the trimerization domain [221]. N-terminal deletions of HSFA1a negatively affect H_2_O_2_ sensing, suggesting that trimerizations are induced by conformational changes [222]. However, the N-terminal deletion of HSFA1a does not inhibit heat sensing. This shows that, despite a common dependency on oxidative activity, the activation of this transcription factor is stress-specifically regulated [217]. The ROS signal has been shown to induce two signaling pathways associated with NO: ROS generated by RbohB and RbohD trigger NO accumulation [126] and the NO signal activates CaM3, which then leads to DNA binding of HSFs [223]. Full activation of HSFA1 seems to involve many processes rising from several signaling pathways [224]. However, recent data have shown that the binding of HSFA1a to its targets can be obtained independently of HS, by altering the plant redox state through light-dependent chloroplast signaling [39]. On the other hand, trimerization of HSFA4A is strictly dependent on the redox environment, since the intramolecular interactions of HSFA4A are weakened when the three conserved Cys residues are replaced with Ala [225,226]. In Arabidopsis, the MAPKs that phosphorylate this HSF, namely MAPK3 and MAPK6, have also been shown to be activated by H_2_O_2_ [225]. These results suggest that HSFA4A can function as redox sensors in stress conditions.

Recently, it has been reported that NO bursts under HS induce S-nitrosylation at Cys324 or Cys347 of the trihelix transcription factor GT-1, which promotes its binding to the HSFA2 promoter (Figure 3). GT-1s loss of function disturbs HSFA2 activation and heat tolerance, revealing that GT-1 is a requested mediator connecting signal perception to the triggering of cellular heat responses [227].

In the HSR, ROS affects nuclear gene expression due to the nuclear translocation of cytoplasmatic transcription factors or associated proteins, subjected to redox-dependent conformational changes (Figure 3). Indeed, cytosol oxidation can trigger the movement of redox-sensitive proteins into the nucleus [179].

HSFA8 shows redox-dependent translocation to nuclei, which is associated with the oxidation of key Cys residues and the formation of disulfide bonds [228]. Mutagenesis of the conserved residues, Cys24 and Cys269, blocks the translocation of HSFA8 to the nucleus, suggesting that this HSF functions as a redox-sensing transcription factor within the stress-responsive transcriptional network (Figure 3).

Cytosolic glyceraldehyde-3-phosphate dehydrogenase (GAPC) is a glycolytic enzyme that is also involved in plant responses to oxidative stress, which could result from abiotic and biotic challenges [229]. One mechanism for the action of GAPC in HSR is stress-induced nuclear translocation [230,231,232]. Under HS, cytosol oxidation triggers GAPC movement to the nucleus, where it interacts with the transcription factor NF-YC10 to promote heat tolerance through the expression of heat-inducible genes [233]. The overexpression of GAPC improves heat tolerance and the effect is removed when NF-YC10 is lacking, heat-induced nuclear accumulation of GAPC is repressed, or the GAPC-NF-YC10 interaction is disrupted. Moreover, the overexpression of GAPC also improves the binding ability of NF-YC10 to its target promoter (Figure 3) [233].

WHIRLY1 (WHY1) is a chloroplast and nucleus-localized plant-specific DNA- and RNA-binding protein that carries out several cellular tasks [234,235]. In the chloroplast, WHY1 mainly works in safeguarding the genome, but it also controls the photochemical activity of PSI [236,237,238]. In the nucleus, WHY1 functions as a transcriptional activator that can bind the elicitor response element of the pathogenesis-related gene PR-10 in Arabidopsis [239,240], or the HvS40 promoter in barley, regulating plant senescence [237]. Recently, a clear association between WHY1 and HSR has been reported. In tomato, HS induces WHY1 expression, and the protein binds the HSE in the promoter of the ER-localized small heat shock protein, HSP21.5A, to activate its transcription (Figure 3). WHY1 overexpression increased thermotolerance; conversely, the inhibition of WHY1 expression leads to an impaired HSR with reduced membrane stability and soluble sugar content and increased ROS accumulation [241]. WHY1 has been proposed as a sensor of photosynthetic apparatus redox changes and represents an ideal candidate for retrograde signaling. In physiological conditions, WHY1 is closely associated with thylakoid membranes. The over-reduction in one or more components of the thylakoid electron transport system, such as the PQ pool/cytochrome b6f complex, may destabilize the oligomeric WHY1 structure on the thylakoid membrane, leading to a release of monomeric proteins, which are then translocated to the nucleus [242].

## 9. Conclusions

Progressively higher temperatures, which are intensified by global warming, may have a strong impact on plant yield in the future. Thus, the study of molecular processes that allow plants to overcome climate change is essential to reduce its negative influence on growth and productivity. Plants use multiple intracellular signaling pathways to sense and respond to high temperature. The effects of heat stress are often correlated with an increase in ROS production occurring in organelles as well as in the apoplast. Even though ROS can potentially cause damage to plant cells, they may also act as signaling molecules and trigger the adaptative response [44,47]. The different and antagonistic role of these reactive molecules depends on a subtle balance between production and scavenging. Therefore, the enhancement of antioxidants is a key strategy to confer heat stress tolerance [53]. However, the study of antioxidant systems in HSR requires further investigation, since antioxidants display a complex and intricate network, which depends on the characteristics of the plant and heat stress, and on the different and sometime antagonistic behaviors of the various antioxidant isoenzymes.

ROS accumulation has been shown to be a requirement for the acquisition of plant thermotolerance. Thus, the research on the sites of ROS generation, the mechanisms that regulate ROS transport, as well as how ROS signaling is interconnected in different compartments is crucial for the comprehension of HS tolerance. The knowledge of the complex dynamics of ROS networks in the different cell compartments can be strongly enhanced using genetically encoded ROS and redox sensors.

Chloroplasts have been shown to play an important role in heat-induced ROS accumulation and the subsequent expression of nuclear heat-responsive genes [129]. Recently, it has been suggested that GUN1, a key player in plastid-to-nucleus retrograde signaling, contributes to the acquisition of basal thermotolerance, being involved in ROS accumulation and oxidation of the cellular environment [113], but, further research is needed to determine the mechanisms involved in this signaling pathway.

Not only HS-induced ROS production, but also HS-related redox changes are strongly linked with the HSR that confers thermotolerance [44,105]. In this context, Cys residues play a significant role as sensors of redox changes, triggering different post-translation modifications, which in turn regulate redox-dependent paths, comprising conformational changes of protein involved in HSR, as well as modifications to transcription factors or associated proteins that can also cause nuclear translocation [179,180,181]. The massive protein regulation capability may strongly affect adaptation to HS. Thus, the continuous advancement in proteomic approaches that permits a more accurate recognition of potential modifications will improve understanding of the significant signaling functions of redox changes occurring in HSR. Moreover, since HS also leads to nuclear oxidation [105], determining the redox regulation of nuclear proteins may help to identify the important mechanisms of genetic and epigenetic regulation of thermotolerance.

The use of model systems and the study of HSR in a controlled environment are crucial to elucidate the redox signaling network activated in response to high temperatures. However, improved knowledge about HSR has to come from experimental where environmental changes need to be simulated as realistically as possible. Indeed, it should be considered that under field conditions, HS is frequently accompanied by other types of stress, such as drought, air humidity, or the presence of pathogens [243,244]. Recent studies reveal that the response of plants to a combination of different abiotic and biotic stresses is unique and cannot be directly extrapolated from simply analyzing each of the different stresses applied individually [103,245,246,247]. Several literature studies have documented the effects of HS combined with other stress factors [160,248,249,250]; nevertheless, comprehensive analyses on the complex network between hormones, ROS, and redox signals in combined stress occurring under complex environmental conditions deserve more attention. Knowledge of how ROS and redox signals combine, and the identification of the numerous compartment-specific redox pathways that are activated might be crucial to modify HSR as a function of HS intensity and duration, and of plant characteristics, in order to help plants deal with climate change.

## Figures and Tables

**Table 1:** List of redox-dependent conformational changes and functions of proteins under heat stress.

Protein	Uniprot Code	Structure and Function		References
		Physiological Conditions	Heat Stress	
AtTrx-h3 (thioredoxin)	Q42403	LMW complexes disulfide reductase activity.	HMW complexes molecular chaperone.	[195]
NTRC (thioredoxin reductase)	Q70G58	LMW complexes disulfide reductase and foldase chaperone.	HMW complexes holdase chaperone.	[196]
2-Cys Prx A (peroxiredoxin)	Q96291	LMW complexes peroxidase activity.	HMW complexes chaperone.	[197]
GRXS17 (Monothiol glutaredoxin)	Q9ZPH2	LMW complexes glutaredoxin involved in the maturation of Fe–S proteins.	HMW complexes chaperone with holdase activity.	[198]
AtNPR1 (Non-expressor of pathogenesis related proteins 1)	P93002	Monomeric form positive regulator of plant systemic acquired response.	Oligomeric form chaperone.	[199]

## Data Availability

No new data were created or analyzed in this study. Data sharing is not applicable to this article.
